# Oral health promotion for schoolchildren – evaluation of a pragmatic approach with emphasis on improving brushing skills

**DOI:** 10.1186/1472-6831-8-4

**Published:** 2008-01-31

**Authors:** Alon Livny, Yuval Vered, Liat Slouk, Harold D Sgan-Cohen

**Affiliations:** 1 Department of Community Dentistry, Hebrew University-Hadassah Faculty of Dental Medicine, Jerusalem, Israel

## Abstract

**Background:**

Preventive dentistry has traditionally emphasized improvement of oral hygiene. School-based programs, often delivered by dental hygienists or other health educators, are usually limited to dental knowledge provision. The present study focused on promotion of health behavior. The objectives were to evaluate the effect of a pragmatic educational program on tooth brushing skills of young schoolchildren.

**Methods:**

The population consisted of 196 first grade children in Jerusalem. One dentist interviewed the children and evaluated base-line brushing skills, applying simple visual index, based on dividing the dentition to eight different segments. a trained hygienist then educated the children, emphasizing brushing skills. A simple "scrubbing" brushing method was taught for all dental surfaces. Four months later a second examination was conducted, applying same evaluation methods.

**Results:**

At base-line 92% of the children had brushed the labial surfaces of front teeth, but only 8% brushed the inner surfaces of posterior teeth. Only 32% brushed occlusal surfaces. These levels significantly increased after four months: 98% now brushed the labial surfaces; 43% brushed inner surfaces of posterior teeth, 87% brushed occlusal surfaces (p < 0.001). The average number of dental "areas" brushed had increased (among the eight areas recorded) from 2.8 to 5.7 (p < 0.0001).

**Conclusion:**

This method of behavioural instruction emphasized improvement of personal manual skills specifically for those areas of the dentition which demand most efforts in oral hygiene promotion. These results are of practical help in improving future health education programs by the health promotion team.

## Background

Health education, traditionally and correctly, one of the cornerstones of preventive dentistry, has over the years involved considerable investments of time, energy, personnel and money. However, there has been a burden of justified criticism, due to lack of evidence-based effectiveness [1,2]. The main reason for this has been the emphasis placed on promoting dental knowledge, which has been demonstrated to have little effect neither on oral health behavior nor on oral health [1]. Oral hygiene, however, has consistently been recognized as the staple and inescapable component of preventing gingivitis and also (although, debatably, to a relatively lesser degree) dental caries [3-7]. A high carbohydrate sweet diet has clearly been implicated in the etiology and epidemiology of dental caries [7]. When efforts are invested in oral health education, it is therefore a rule of thumb that emphasis should include dietary and oral hygiene improvement [7,8].

Dental hygienists are the conventional but not the only primary agents for improving oral hygiene [9]. The Jerusalem municipality, under the auspices of the Israeli Health Ministry, is responsible for a city program aimed at oral health promotion in primary schools. This program, conducted by dental hygienists, has focused on health education, and has included a long list of educational themes such as basic dental anatomy, functions of teeth, good *vs*. bad foods, the role of bacteria, dental plaque, etc. Tooth brushing skills are usually taught to an entire class of children, employing a large brush and "dental mouth" model.

A wide range of tooth brushing methods have been described and evaluated. These demand considerable manual dexterity and include brushing with specific directions, movements and angles between the brush, the gingival tissue margins and the teeth. Among young children an easy and adequately effective "horizontal scrubbing" method has often been advocated and employed [10].

The manual skills of tooth brushing, and improvement of these at the personal level, are traditionally evaluated by means of plaque removal success, and measured by plaque scores and indices [10-12]. These indices are time consuming, demand professional expertise and training and often include application of colored plaque disclosing solutions, which are difficult to employ in regular school settings. Simmons [13] has developed an index for evaluating "brushing skills", operationally defined as location of toothbrush positioning in the mouth and those dental surfaces reached, during habitual brushing. This index is comprised of counting and coding eight different locations of the dentition, which are brushed by subjects in the presence of the examiner. The index does not measure the quality of brushing and the effective removal of dental plaque.

The purpose of the present study was to assess the feasibility and the effectiveness of a modified oral health promotion program, based primarily on improving tooth-brushing skills and conducted over three weeks by a dental hygienist.

The specific objectives included: 1. to measure the proportion of children who brushed each of the eight locations before and after the program; 2. to assess effect of the program on reported daily brushing frequency; 3. to assess effect of the program on reported dietary behavior; 4. to ascertain who were the main sources of tooth brushing and oral hygiene guidance.

## Methods

The sample size was calculated according to a previous study, employing the same method [13]. Based on these data, for detecting a 20% increase in brushing frequency, for a power of 95% and a significance level of 5%, a minimal sample of 144 children was required [14].

Five primary schools were purposively chosen in those Jerusalem neighborhoods where the municipal dental hygienist was currently working. In nine first grade classes, 227 children were enlisted (an average of 25 children per class). The study population was classified (according to municipal records) as including medium-low socio-economic levels, religious and secular Jewish families.

This was a "whole population" sample and all children (who were currently being educated by the hygienist) participated. The study was incorporated within the ongoing municipal health education program (by law and according to Israeli Ministry of Health guidelines) and therefore required no additional IRB (Internal Review Board for human experiments ethical committee) approval and parental informed consent.

At base-line, the one dentist examiner (L.S.) interviewed each child (see appendix) as to: diet brought to school that day (sandwiches with sweetened or non-sweetened sandwich spreads, sweetened soft drinks or mineral water, fruit brought to school; number of times a day that teeth were usually brushed (never, once, twice, thrice, or more); and who had taught the child to brush teeth (parents, school nurse, dentist, hygienist, teacher, other).

The dentist then gave the child a toothbrush (distribution of brushes and paste are a regular component of the municipal program) and requested that the child demonstrate how he/she brushes. The dentist recorded which of the eight locations were brushed, according to the Simmons "Brushing Skills" index [13], which records the following eight locations:

- A = buccal surfaces of maxillary and mandibular front teeth (central and lateral incisors and canines)

- B = buccal surfaces of maxillary posterior teeth (premolars and molars)

- C = buccal surfaces of mandibular posterior teeth

- D = occlusal surfaces (of posterior teeth)

- E = lingual surfaces of maxillary front teeth

- F = lingual surfaces of mandibular front teeth

- G = lingual surfaces of maxillary posterior teeth

- H = lingual surfaces of mandibular posterior teeth

Due to the on-going "field" nature and restraints of this program it was difficult to conduct intra-examiner calibrations. The dentist examiner had been trained by and calibrated with the last author (H.S.C.). Agreement levels had reached more than 90% and were considered adequate.

Approximately one week later, the dental hygienist commenced the three week (one lesson per week) dental health education program. In contrast to the previous lesson guidelines, she was instructed to place emphasis on brushing skills. Most of the time was therefore spent on individual training of the children, supervised brushing of all dental locations, tailored to each child's observed skills, and verification that each of the eight areas was brushed. The remaining time included explaining the use of fluoridated toothpaste and healthy dietary habits. Further topics were purposively excluded from these lessons.

Four months later the dentist revisited the children and collected the same data (interview and observation of brushing skills) as at base-line.

Statistical analyses, comparing the two examinations, took into account the pair-matched structure of the data (the same children before and after). The Chi Square test was employed in comparison of sandwiches (sweetened or non-sweetened spreads), drinks (sweetened or non-sweetened), fruit (yes or no) and regular tooth brushing frequency (once or less, twice or more times a day), before and after the program. The McNemar's test was also used to compare the percent of children who brushed each dental location before and after the program. The p values were adjusted for multiple testing by means of the Holms procedure. Wilcoxon Rank test was used in comparison of number of locations brushed before and after the program. Subgroup analysis of gender, as a possible confounder, was performed by stratifying data separately by this variable. Statistical level of significance was chosen at p < 0.05.

## Results

Of the original study population of 227, 31 children (13%) dropped out due to absence from school at one of the two examination days (19 boys, 12 girls). The 196 remaining children were equally distributed by gender.

### Reported diet

Before the program, 37.7% of all the children reported bringing sandwiches with sweetened spreads (chocolate spread, jelly, etc.). This level decreased to 33.2% after the program (not statistically significant). A significant decrease in sweet sandwiches was found separately for boys: from 46.8% to 29.6% (p = 0.003).

Before the program, 39.7% of all the children reported bringing fruit to school. This increased to 53.9% after the program (p = 0.001). When separating by gender, boys increased bringing fruit from 34% to 46.4% (p = 0.003), but no significant difference was found for girls.

Before the program, 22.4% of the children reported bringing sweetened soft drinks to school. This level decreased to 13.3% after the program (p = 0.01). Here, too, boys showed a marked decrease from 24.4% to 11.2% (p = 0.002), but no significant difference was found for girls.

### Reported tooth brushing practices

As shown in Table 1, before the program 67.2% of the children reported brushing once a day and 32.8%% twice a day. This improved after the program to 12.6% once a day and 97.4% twice a day (p < 0.0001). No children had reported brushing more than twice a day. Two had reported never brushing, before the program, but brushed once a day afterwards. The significant improvements were the same for both genders.

**Table 1:** Reported brushing frequency of children per day – before and after the program (N = 189)

		After program		
		once	twice	total
		
before program	once	21	106	127 (67.2%)
	twice	3	59	62 (32.8%)
	total	24 (12.6%)	165 (97.4%)	189 (100%)

Chi Square test (continuity corrected), p < 0.0001 (for males, females and total) missing data from seven children

As for the source of their brushing skills, most children (88.7%) reported having been taught by their parents, and only 8.2% by their dentists. Negligible numbers (3.1%) reported other sources.

### Improvement of brushing skills by location

As is shown in Table 2, at base-line, the vast majority of children brushed only their labial anterior segments (92.3%). Brushing of the lingual surfaces of teeth was rare (8–10%). After the program, a statistically significant increase was noted for all surfaces. Improvements were highly significant for both genders, except for the labial anterior segment, where the proportion increase was significant for girls (p = 0.02) but not for boys. After the program the majority of children now brushed most locations. The exceptions were the lingual (maxillary and mandibular) posterior surfaces, which were brushed by only 43.1% of the children after the program. It should be noted that these surfaces had been brushed by the smallest proportions of children before the program (8%).

**Table 2:** Percentage of children demonstrating brushing skills by dentition segments, before and after program

segment	N	before	after	difference	95% Confidence Interval
A – labial anterior	196	92.3%	98.5%	6.2%	3.4 – 10.0
B – buccal maxillary	196	63.8%	94.4%	30.6%	24.5 – 37.3
C – buccal mandibular	196	61.7%	92.9%	31.1%	24.9 – 37.9
D – occlusal	193	32.1%	87.0%	54.9%	47.9 – 61.8
E – lingual maxillary anterior	194	10.3%	61.9%	51.6%	44.5 – 58.5
F – lingual mandibular anterior	195	9.7%	55.9%	46.2%	39.2 – 53.2
G – lingual maxillary posterior	196	8.7%	43.4%	34.7%	28.3 – 41.6
H – lingual mandibular posterior	195	8.2%	43.1%	34.9%	28.4 – 41.8

Statistical analysis by McNemar test, comparing yes/no before *vs*. yes/no after, adjusted by the Holms method for multiple testing:

A: for males and total not significant, for females: p = 0.002

B-H: for males, females and total: p < 0.001

Missing data from 1–3 children, for segments: D, E, F, H

Table 3 demonstrates that the mean number of brushed locations (out of the total potential eight) increased approximately two-fold after the completion of the program – for boys, girls and total population.

**Table 3:** Average number of dental segments brushed before and after program (N = 196)

	Males (N = 98)		Females (N = 98)		Total (196)	
	
	mean	SD	mean	SD	mean	SD
before	3.15	1.92	2.57	1.67	2.86	1.82
after	5.88	2.35	5.64	2.08	5.76	2.21
difference	2.73		3.07		2.90	
95% C.I.	2.27 – 31.8		2.66 – 3.48		2.59 – 3.20	

According to Wilcoxon Rank test, in comparison of number of locations brushed before and after the program: p < 0.0001 (for males, females and total)

## Discussion

Oral health promotion in primary school settings has traditionally focused on improving dental knowledge. The aim of this study was to evaluate the employment of a pragmatic, simple and potentially effective approach, focusing on oral hygiene practices, and improving manual tooth brushing skills. This presented a challenge to the tradition-based rationale of imparting an abundance of information, without any measurable evidence-based effect.

This study employed a previously used and validated tool for measuring "brushing skills" [13], which showed that most preschool children primarily brushed labial anterior surfaces, and to a lesser degree all other segments. The present results were consistent with these previous findings. A similar and improved pattern was identified among first grade schoolchildren.

It could have been assumed that young children would present manual dexterity difficulties in brushing lingual and even occlusal surfaces of teeth. However, even for lingual surfaces, this study demonstrated significant improvements in brushing skills. The low proportion of children who initially brushed their occlusal surfaces (only 32.1%) was specifically disturbing, as this is the age of highest risk for occlusal "pit and fissure" caries initiation. Occlusal caries in pits and fissures is the form of caries characterized by the earliest onset, with the most extreme potential harm accumulating over life (ultimately often leading to loss of teeth). The significant increase in the proportion of children brushing these surfaces (32.1% to 87%, a 2.7 ratio increase), after the present program, supplied substantial justification for the present approach.

There was also a significant improvement in reported dietary behavior, specifically for the boys in these classes. Albeit diet was assessed by interview and therefore response bias could have been suspected, it should be noted that the question pertained to what had been brought to school "today", and therefore, children knew that the dentist examiner could, and often did, objectively validate these reports. Since not much time was dedicated to dietary behavior, we tend to relate this finding to the general improvement of dental awareness and attitudes in the classes during the program. It was beyond the realm of this study to explain the difference found by gender. Boys appeared *a priori *to demonstrate poorer dietary habits and therefore potential improvement was more easily possible.

The literature has presented a consensus that tooth brushing, at least once a day is essential for maintenance of oral health, prevention of caries and periodontal diseases, and is an important vehicle for application of fluorides. It is acknowledged that most people do not practice optimal plaque removal and therefore tooth brushing is commonly recommended twice daily [15].

Most children in this study (88.7%) reported that their parents were the major source of oral hygiene guidance. Programs should, therefore, include the involvement of parents who are integrally related to children's health behavior. Only few children reported to have been taught by dentists, and none by dental hygienists or other health personnel. This finding certainly indicates that health educators in the present setting have a potentially important and presently unfulfilled role [9].

The present study population cannot be regarded as a representative sample of the whole of Jerusalem. The population, however, was considered as heterogeneous and included most of the common Jerusalem socio-economic groups.

This was not a randomized controlled trial (RCT) and did not include a control group. The problem of dependent multiple observations (before and after) of the same subjects has been related to in the literature [16]. This might have influenced and biased the measured treatment effect in the present study. We were unable to estimate the magnitude of this potential bias and carry-across effect. We believe that the one examiner could not have remembered individual children four months later, but could have been biased, by knowing that the whole group had participated in the program. An optimal RCT, including a no-treatment control group, very likely, might not have been approved by the IRB.

The non-compliers (13%) might also be considered a source of potential bias (although their absence from school at the day of data collection cannot be considered as intentional). Other methodological problems that should be addressed are the lack of intra- or inter-examiner (more than one examiner) calibration, the self-reported evaluation of daily brushing frequency, and the fact that children were aware of being watched while brushing. These factors could have raised problems of potential examiner and examinee (social desirability) biases. Regarding the potential examiner bias, we assume that the dentist possibly may have amplified the general improvement after the program, but less so the differentiated changes for each specific dental location. Similarly, we believe that the potential examinee biases could have influenced the general self-report of brushing, but would not have explained the different levels of specific brushed locations, as observed by the dentist.

The aim of the present study was to examine and demonstrate a potential mode of improving tooth brushing skills. This study did not measure effectiveness in terms of expected clinical outcomes: plaque removal or reduction of caries and gingivitis. There were certain design limitations and this was not an optimal RCT. It was not possible to determine what other oral hygiene instruction from additional sources (media, private dental hygienists and dentists etc.) may have been invested at the same time and could have confounded the results. The observation period was only four months, which might be too short to measure long-term effects. Despite these limitations, the results clearly demonstrated the positive program effect on brushing skills.

Dental hygienists, school nurses and other health personnel regularly visit schools and offer oral health education. In Jerusalem, hygienists visit schools on an annual basis. The previous program included delivering a large body of dental health knowledge. The modified program described in this study could consist of fewer sessions per class, and therefore might potentially cover more children, more frequently, each year.

## Conclusion

Dental caries is inescapably the most prevalent disease of young children in most countries. Preventive oral health programs in the past have been commonly unbeneficial. Due to the present study's proposed simplistic pragmatic approach, this program could be delivered by general health educators and not only dental hygienists. This reorientation of the health promoting workforce, together with the development of personal skills at young ages, are two of the cornerstones of health promotion as stated in the Ottawa Charter [17]. The potential advantage to young schoolchildren of this approach is evident and should be further explored in the future over longer than four month periods.

## Competing interests

The author(s) declare that they have no competing interests.

## Authors' contributions

AL was co-researcher in this study, and the main author of this manuscript. YV contributed to the writing and contributed to the statistical analyses, LS was the main field researcher and carried all the data collection. HSC conceived the study, and supervised all its stages.

## Appendix

### Interview questionnaire for each child

1. Date

2. School

3. Class

4. Male/Female

5. Description of sandwich brought today to school, specifying sandwich spread (open end question)

6. Was fruit brought today to school? YES/NO

7. Description of drink brought today to school, specifying: WATER/SWEETENED SOFT DRINK/OTHER (specify if "other")

8. How many times a day do you usually brush your teeth? NEVER/1/2/3/more

9. Who taught you to brush your teeth? SCHOOL NURSE/DENTIST/PARENTS/HYGIENIST/TEACHER/OTHER (specify if "other")

## Pre-publication history

The pre-publication history for this paper can be accessed here:


